# American Medical Association

**Published:** 1849-02

**Authors:** 


### AMERICAN MEDICAL ASSOCIATION.

At the request of the Secretaries of the American Medical Association, we invite the attention of our readers to the following notice, which we find in the *Philadelphia Medical News and Library* for January.

"By desire of the committee of arrangements, the Secretaries of the American Medical Association request that all societies and other institutions, authorized to appoint delegates, send correct lists of those chosen to attend the next annual meeting, to Dr. Henry J. Bowditch, Boston, on or before the 1st of April, 1849.

"We would invite attention to this request, as a compliance with it will greatly facilitate the organization of the Association.

"We take this opportunity to remind the members of the Association of the resolution adopted in Baltimore, directing that a copy of the Transactions should be sent to such members only as shall have paid the annual assessment for the present year (three dollars). Those members paying to the Treasurer five dollars, are entitled to three copies.

"Medical Societies which have been represented in the Association, will be furnished copies on the same terms as members, (viz: three copies for five dollars) on remitting the amount to the Treasurer.

"To other persons, the Transactions will be furnished at the rate of two dollars per copy in paper covers, done up for mail, or two dollars and fifty cents in embossed cloth, on remitting the amount direct to Messrs. Lea & Blanchard, Philadelphia. Or orders left with booksellers, will be executed by Messrs. Lea & Blanchard."

